# Intra-Tumoral Lymphocytic Infiltration Is Associated with Favorable Prognosis in Suboptimal Surgery in High-Grade Serous Ovarian Carcinoma

**DOI:** 10.3390/diagnostics15040422

**Published:** 2025-02-10

**Authors:** Hiroshi Harada, Toru Hachisuga, Yoshikazu Harada, Mami Shibahara, Midori Murakami, Fariza Nuratdinova, Shota Higami, Atsushi Tohyama, Yasuyuki Kinjo, Taeko Ueda, Tomoko Kurita, Yusuke Matsuura, Toshiyuki Nakayama, Kiyoshi Yoshino

**Affiliations:** 1 Department of Obstetrics and Gynecology, School of Medicine, University of Occupational and Environmental Health, 1-1, Iseigaoka, Yahatanishi-ku, Kitakyushu 807-8555, Japan; thachisu@med.uoeh-u.ac.jp (T.H.); shibahara-mami@med.uoeh-u.ac.jp (M.S.); m-midori@med.uoeh-u.ac.jp (M.M.); s-higami0501@med.uoeh-u.ac.jp (S.H.); a-tohyama@med.uoeh-u.ac.jp (A.T.); kinjo-yasuyuki@med.uoeh-u.ac.jp (Y.K.); uedataeko@med.uoeh-u.ac.jp (T.U.); t-kurita@med.uoeh-u.ac.jp (T.K.); k-yoshino@med.uoeh-u.ac.jp (K.Y.); 2 Department of Pathology, School of Medicine, University of Occupational and Environmental Health, 1-1, Iseigaoka, Yahatanishi-ku, Kitakyushu 807-8555, Japan; harada-y@med.uoeh-u.ac.jp (Y.H.); fariza.nuratdinova@gmail.com (F.N.); toshi-n@med.uoeh-u.ac.jp (T.N.); 3 Department of Nursing of Human Broad Development, School of Health Sciences, University of Occupational and Environmental Health, 1-1, Iseigaoka, Yahatanishi-ku, Kitakyushu 807-8555, Japan; yusuke-m@med.uoeh-u.ac.jp

**Keywords:** high-grade serous ovarian carcinoma, tumor-infiltrating lymphocytes, immunoreactive subtype, suboptimal surgery, cancer immunity, prognosis

## Abstract

**Background:** The immunoreactive (IR) subtype is one of the subtypes of high-grade serous ovarian carcinoma (HGSOC) with intra-tumoral lymphocytic infiltration. A positive prognostic correlation between IR subtype and R0 + optimal surgery has been reported. This study investigates the prognostic significance of tumor-infiltrating lymphocytes (TILs) in the suboptimal surgery group of HGSOCs. **Methods:** After reviewing 318 malignant ovarian tumors detected in our database between 2000 and 2017, 74 HGSOCs with supplemental p53 immunostaining were selected. Differences in progression-free survival (PFS) and overall survival (OS) between the two groups of the IR subtype and the other subtypes were investigated. Based on pathological findings, HGSOCs were divided into two groups: those with or without abundant TILs. **Results:** Abundant TILs were detected in 17 cases of HGSOC (22.9%). Clinicopathological characteristics including age, CA125, FIGO stage, and residual disease did not show significant differences between the two groups. Lymph node metastasis was more common in the IR subtype group (*p* = 0.04). In the suboptimal surgery group, the 5-year PFS and OS (Kaplan–Meier estimates) in cases with (n = 10) or without (n = 21) abundant TILs were 10% and 0% (*p* = 0.097) and 70% and 28.5% (*p* = 0.012), respectively. The median time (range) to OS in cases with or without abundant TILs were 58 (34–81) months and 39 (22–55) months, respectively. Multivariate analysis in OS showed significant differences in TILs. **Conclusions:** Abundant intra-tumoral lymphocytic infiltration is an independent and favorable prognostic indicator for the suboptimal surgery group in HGSOCs and is associated with treatment response via cancer immunity.

## 1. Introduction

High-grade serous ovarian carcinoma (HGSOC), the most common histological type of epithelial ovarian cancer, is often diagnosed at an advanced stage and is associated with a high risk of recurrence and a poor prognosis. In cytoreductive surgery and adjuvant treatment for advanced cancer, R0 (complete resection) or optimal surgery (residual tumor less than 1 cm gross) may improve prognosis, whereas suboptimal surgery (residual tumor above 1 cm gross) is associated with poor prognosis [1]. HGSOC can be classified into four molecular subtypes: immunoreactive (IR), differentiated, proliferative, and mesenchymal, identified by transcriptomic profiling [2]. The IR subtype of HGSOC is characterized by abundant tumor-infiltrating lymphocytes (TILs) in the tumor microenvironment and a high expression of immune-related genes, and it tends to have a better prognosis compared to other subtypes [3,4,5,6]. The improved prognosis of the IR subtype may be due to higher intratumor immune activity, which helps to eliminate cancer cells more effectively; studies have shown that the IR subtype is associated with longer overall survival (OS) and progression-free survival (PFS) [2,3,4,5,6]. The combination of a high presence of TILs with R0 or optimal surgery is a favorable prognostic indicator. However, for patients who have undergone suboptimal surgery, the utility of TILs in predicting outcomes is still unclear. This study investigated the prognostic significance of TILs in patients treated with R0 + optimal and suboptimal surgery for HGSOC.

## 2. Materials and Methods

**Figure 1:**
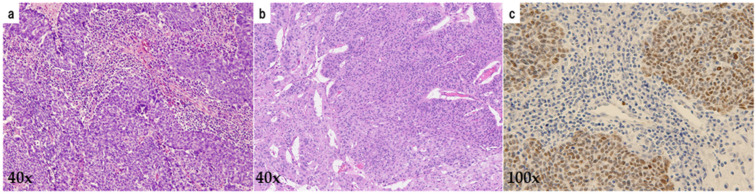
Histopathological images of HGSOC. High-grade serous ovarian carcinoma with abundant infiltrating lymphocytes within the tumor and stroma on Hematoxylin-Eosin (H&E) Staining (**a**). High-grade serous ovarian carcinoma without abundant tumor-infiltrating lymphocytes (**b**). p53 stained section showed aberrant overexpression (**c**).

**Figure 2:**
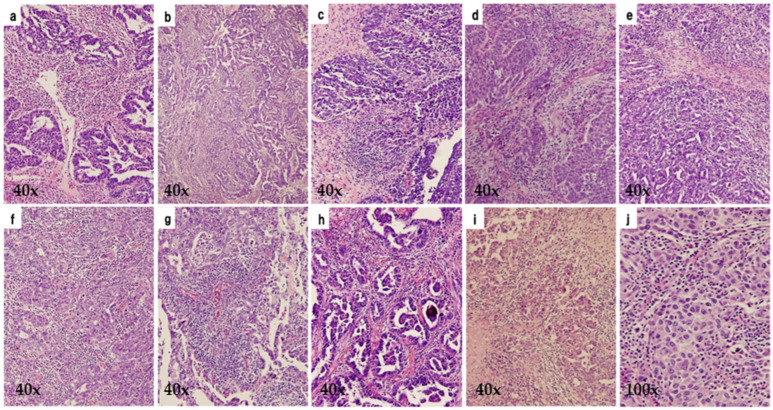
Histopathological images of 10 HGSOCs with IR presence (IR subtype). The 10 cases of high-grade serous ovarian carcinoma with abundant tumor-infiltrating lymphocytes in the suboptimal surgery group (**a**–**j**).

We reviewed 318 malignant ovarian tumors detected in our database between 2000 and 2017. A total of 74 HGSOCs were selected histopathologically with supplementary p53 immunostaining to exclude low-grade serous ovarian carcinoma. Histopathological images are shown in Figure 1. Paraffin-embedded specimens were obtained from the archives of the University of Occupational and Environmental Health, Japan, and relevant clinical data were also collected for analysis. The selection of diagnostic criteria and stratification factors for HGSOC was based on the International Federation of Gynecology and Obstetrics (FIGO) cancer report and the consensus conference recommendations of the European Society of Gynecological Oncology (ESGO), the European Society for Medical Oncology (ESMO), and the European Society of Pathology (ESP) or the GCIG (Gynecologic Cancer Intergroup) [1,7,8]. The following factors were extracted from 74 cases: age at diagnosis, FIGO 2014 stage, initial surgical treatment (primary debulking surgery or interval debulking surgery), retroperitoneal lymph node sampling, initial platinum-based chemotherapy (neoadjuvant or adjuvant), CA125 level before initial treatment, residual disease after surgery (R0, optimal surgery, suboptimal surgery), recurrent treatment, architectural grade, nuclear grade, lymphovascular space invasion, intra-tumoral budding, p53 expression status, HGSOC histological subtypes (immunoreactive, differentiated, proliferative, and mesenchymal), tumor-infiltrating lymphocytes (TILs), overall survival (OS), and progression-free survival (PFS). Response to chemotherapy was defined according to RECIST (Response Evaluation Criteria In Solid Tumors) [9]. TILs and IR subtype were assessed concerning a previously published algorithm [4]. The mesenchymal transition subtype was diagnosed when tumor cell clusters with a labyrinthine structure or cells lacking continuity infiltrating with stromal reaction were present in more than 10% of the slides. In cases without MT features, TILs were counted for localization within or outside cancer nests using one representative slide from each case at 400× magnification; the number of TILs was the median value obtained from the 5 most heavily infiltrated areas. Tumor nests with abundant TILs (>100/×400 fields of view) were classified as the IR subtype; those with poor TILs were classified as other subtypes (solid and proliferative type or papilloglandular type). Based on pathological findings, HGSOCs were divided into 2 groups: those with or without abundant TILs. The differences in PFS and OS between the 2 groups of IR subtype and the other subtypes of HGSOC were investigated. Histopathological images of 10 cases diagnosed as IR subtypes are shown in Figure 2. The group with histopathological evidence of abundant TILs was defined as High TILs (IR subtype) and the group without abundant TILs as Low TILs (the other subtypes). The 2 gynecologic oncologists (T.H. and H.H.) reviewed H&E slides independently to classify the HGSOC subtype. When their histopathological diagnoses were different, the decisions were made by discussion (T.H. and H.H.).

A total of 74 representative formalin-fixed paraffin-embedded (FFPE) tissue blocks were selected for immunohistochemical analysis. p53 immunohistochemistry was performed using a commercially available mouse monoclonal anti-human antibody (protein clone DO-7, Agilent, Santa Clara, CA, USA) at a 1:50 dilution on formalin-fixed paraffin-embedded tissue sections. Sections were stained using the universal immunoperoxidase polymer method (Envision kit; Dako, Glostrup, Denmark) according to the manufacturer’s instructions. Antigen retrieval was then performed by microwave heating and pressure cooking. Positive and negative controls were also performed. According to the proposed immunohistochemical scoring, p53 expression was scored as overexpression or complete absence.

Statistical analyses were performed using IBM SPSS Statistics, version 28 (IBM SPSS Statistics for Windows, IBM Corporation, Armonk, NY, USA). Data on clinicopathological factors were evaluated using the chi-squared test or the Mann–Whitney U test. Progression-free survival (PFS) was defined as the time from the date of initial treatment to the date of objective disease progression or last follow-up. Overall survival (OS) was defined as the time from the date of initial treatment to the date of death or last follow-up. PFS and OS curves were estimated using the Kaplan–Meier method and compared using the log-rank test. Univariate and multivariate Cox proportional hazards models were used to examine the association of potential risk factors with disease progression and death. Statistical significance was defined as a *p*-value of <0.05.

Ethical approval for this study was granted by the Ethical Review Committee of the University Hospital for Occupational and Environmental Health, Japan (UOEHCRB21-155). The ERC waived the requirement for written informed consent because of the retrospective nature of the study [10]. All procedures were carried out according to relevant guidelines and regulations.

## 3. Results

### 3.1. Characteristics of HGSOC in 74 Cases

In terms of clinical background, a comparison between the two groups with high or low TILs showed no obvious significant differences, except for a significant trend in the residual tumor category in Table 1. Similar comparisons of histopathological background between the two groups showed that more cases with low TILs had low-grade structural atypia and more cases with negative lymph node metastasis, and there were no obvious significant differences in the other parameters in Table 2. Although the authors have previously reported on intra-tumoral budding and prognosis in HGSOC, no correlation between intra-tumoral budding and TILs was found in the current study [11].

### 3.2. Kaplan–Meier Survival Curve and Analysis (High TILs Versus Low TILs)

**Figure 3:**
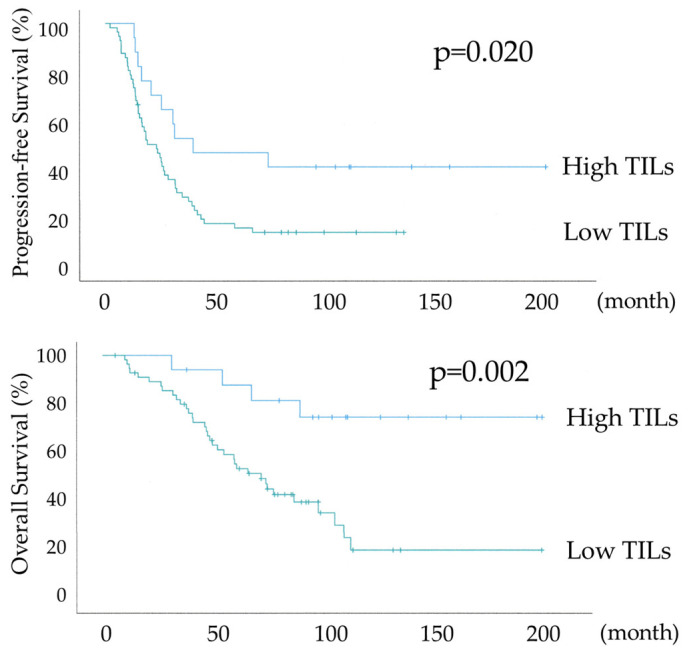
Kaplan–Meier survival curve analyses of the progression-free survival and overall survival in all 74 cases of high-grade serous ovarian carcinoma with or without abundant tumor-infiltrating lymphocytes (High TILs: 17, Low TILs: 57).

Figure 3 shows the differential Kaplan–Meier PFS and OS curves in all 74 HGSOC cases for the high or low TILs. The median PFSs were 38 months (range 28–93) in 17 patients with HGSOC with high TILs and 22 months (range 15–28) in 57 women with HGSOC with low TILs, and their median OSs were not available and 68 months (range 49–86), respectively. Both PFS and OS were significantly prolonged in the high TILs group (*p*-values = 0.020 and 0.002, respectively), with an overall survival of >70% at 120 months in the high TILs group.

**Figure 4:**
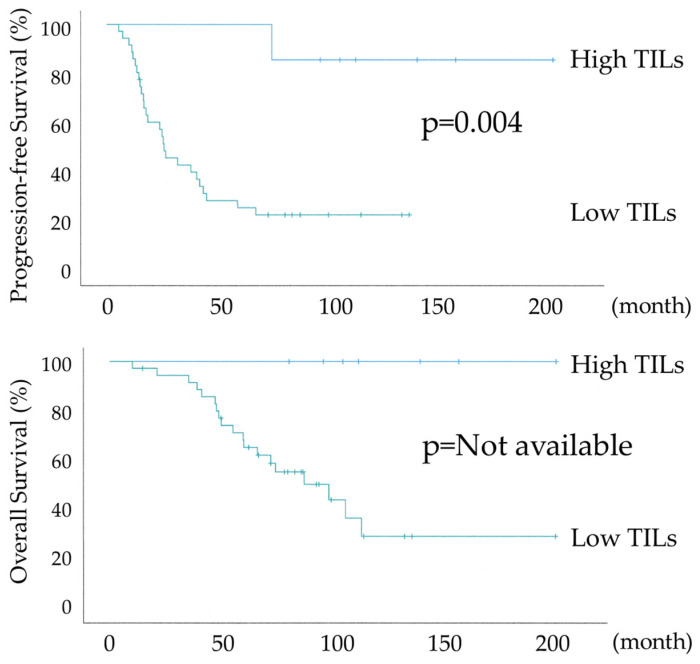
Kaplan–Meier survival curve analyses of the progression-free survival and overall survival in R0 + optimal surgery group with or without abundant tumor-infiltrating lymphocytes (n = 43, High TILs: 7, Low TILs: 36).

Figure 4 shows the differential Kaplan–Meier PFS and OS curves in c for the high or low TILs. The median PFSs were not available in 7 patients with HGSOC with high TILs and 24 months (range 15–32) in 36 women with HGSOC with low TILs. Both PFS and OS were significantly prolonged in the high TILs group (*p*-values = 0.004 and not available, respectively). The high TILs group had >80% PFS and 100% OS at 120 months.

**Figure 5:**
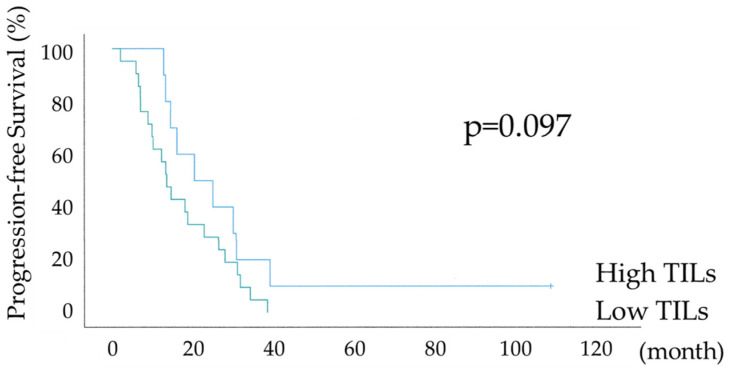

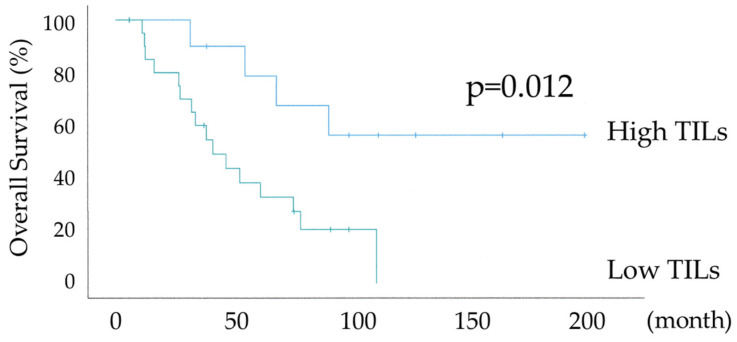
Kaplan–Meier survival curve analyses of the progression-free survival and overall survival in the suboptimal surgery group with or without abundant tumor-infiltrating lymphocytes (n = 31, High TILs: 10, Low TILs: 21).

Figure 5 shows the differential Kaplan–Meier PFS and OS curves in the suboptimal surgery group for the high or low TILs. The median PFSs were 20 months (range 7–32) in 10 patients with HGSOC with high TILs and 13 months (range 10–15) in 21 women with HGSOC with low TILs, and their median OSs were 58 months (range 34–81) and 39 months (range 22–55), respectively. PFS showed a significant trend, whereas OS was significantly prolonged in the High TILs group (*p*-values = 0.097 and 0.012, respectively). In the high TILs group, >50% OS was observed at 120 months. The lack of a significant difference in PFS is because the suboptimal surgery group had a higher risk of recurrence, as the gross residual tumor size at surgery was >1 cm.

### 3.3. Clinical Data of 10 HGSOCs with High TILs in the Suboptimal Surgery Group

Regarding initial treatment, six patients received primary debulking surgery (PDS) and four patients received neoadjuvant chemotherapy (NACT) + interval debulking surgery (IDS). RECIST evaluation after initial treatment showed an overall response rate of 100%, with nine cases as CR and one case remaining relapse-free. Platinum-based chemotherapy was used to treat recurrence in seven cases and radiotherapy to control local recurrence in three cases. In only one case was a poly ADP-ribose polymerase (PARP) inhibitor used to treat recurrence, as the patient was diagnosed with HGSOC before 2017. In terms of prognosis, seven cases had a post-treatment survival of more than 5 years.

### 3.4. Univariate and Multivariate Survival Time Analyses for PFS and OS

In the univariate analysis of PFS, significant differences were found in seven categories, as shown in Table 3. Multivariate analysis showed no significant differences except for the category of residual tumor at surgery (R0 + optimal vs. suboptimal), with a significant trend towards the TILs category. In the univariate analysis of OS, significant differences were found in five categories, as shown in Table 4. Multivariate analysis showed significant differences in the categories of CA125 levels before initial treatment, TILs, and residual tumor at surgery. Regarding the content of the analysis, it is well-known that residual tumor at surgery has an impact on prognosis, and TILs functioned as an independent prognostic indicator in OS.

## 4. Discussion

The initial treatment for advanced epithelial ovarian cancer is primary debulking surgery (PDS) to remove as much tumor as possible to eliminate residual disease, followed by platinum-based chemotherapy and maintenance therapy to prevent recurrence [1]. In relapse, the choice of the chemotherapeutic agent is also based on the presence or absence of platinum sensitivity, and relapse treatment regimens include single-agent platinum chemotherapy, platinum combination, pegylated liposomal doxorubicin (PLD), topotecan, gemcitabine, taxanes, etoposide, and other agents [1,12]. However, patients with advanced epithelial ovarian cancer often have unresectable disease or medical complications that preclude primary debulking surgery. If the tumor cannot be removed with initial surgery, neoadjuvant chemotherapy (NACT) is administered to reduce the tumor size, followed by interval debulking surgery (IDS) to remove the tumor, and thereafter chemotherapy and maintenance therapy are continued. While suboptimal surgery (residual tumor ≥ 1 cm) has a high risk of recurrence and a poor prognosis, complete resection (R0) or optimal surgery (residual tumor < 1 cm) is recommended [1]. PDS and NACT + IDS have been compared in several clinical trials, with three trials showing non-inferiority of NACT + IDS, although NACT + IDS has a poor prognosis when complete resection cannot be achieved [13,14,15,16,17]. Achieving R0 or optimal surgery in PDS and R0 in NAC + IDS is associated with a longer prognosis. Meanwhile, based on genomic analysis of The Cancer Genome Atlas (TCGA) and histopathological findings, several reports have identified IR subtypes of HGSOCs and examined their prognostic value. [2,3,4,5]. The IR subtype is considered to have a better prognosis than the other subtypes; furthermore, the IR subtype tends to achieve R0 or optimal surgery more frequently and is reported to have a better prognosis than the other subtypes. However, in HGSOCs undergoing suboptimal surgery, the prognostic relevance of IR or the other subtypes has not been clarified.

As shown in Table 1 and Table 2, the IR subtype group tended to have more structural atypia and more cases of lymph node metastasis, with a bias towards oncological poor prognostic factors. Nevertheless, as shown in Figure 3 and Figure 4, PFS and OS were significantly prolonged in the IR subtype group in all 74 cases and R0 + optimal surgery, and in Figure 5, significant prolongation in OS was observed in the IR subtype even in suboptimal surgery. Table 3 shows 100% overall response (OR) rates after initial treatment in the IR subtype group with suboptimal surgery. The majority of cases were CR. Although the suboptimal surgery group has a poor prognosis due to high tumor residuals, 7 out of 10 patients in the IR subtype had a survival rate of more than 5 years. Despite multiple recurrences due to residual tumors at surgery in many cases, the prognosis for the IR subtype was maintained due to the high treatment response to recurrence. In univariate and multivariate analyses of all 74 cases shown in Table 4, residual tumor after surgery was an independent prognostic factor for PFS and OS. There was a significant trend towards TILs in the multivariate analysis for PFS and a statistically significant difference in the multivariate analysis for OS, with TILs being the prognostic indicator.

TILs have been studied for decades, and high levels of intra-tumoral lymphocytic infiltration have been reported as a good prognostic biomarker [6,18]. Recent findings on the existence of immune surveillance mechanisms against cancer, cancer cell proliferation through cancer immune escape, and cancer immune subcycles in the tumor microenvironment have led to reports that TILs are involved in maintaining effector phase function [19,20,21,22]. In particular, CD8-positive T cells correlate with prolonged survival [18,23,24]. Elimination of cancer cells requires activation or de-repression of T cell function, and a state of impaired immune function known as immune desert has been reported to reduce the efficacy of anticancer drug treatment [22]. TILs have been reported to be associated with the immunotherapeutic response, including primary and secondary immunotherapy in other cancers, suggesting a link with cancer immunity [25,26,27]. There are also reports that low lymphocyte counts due to cachexia tendency and poor nutritional status suppress cancer immunity [28]. Cancer immune responses are also involved in immunogenic tumor cell death after radiotherapy, including the abscopal effects [29,30,31]. Thus, the importance of the cancer immunity cycle in treatment has been widely recognized since the advent of immune checkpoint inhibitors (ICI) [21,23]. The presence of TILs improves the response to treatment with anticancer agents such as platinum drugs or radiotherapy, leading to a favorable prognosis. A previously established histopathological subtyping scheme to classify HGSOC into four subtypes showed that the IR subtype, corresponding to the TCGA immunoreactive type, had the best prognosis, whereas the mesenchymal transition subtype, corresponding to the TCGA mesenchymal type, had the worst prognosis [2,4]. A study of TILs in HGSOC using artificial intelligence also reported longer OS and PFS in the TIL-high group compared to the TIL-low group [6]. In another analysis of HGSOC subtypes, other subtypes with inferior tumor immune response show PD-L1 regulation and increased tumor growth in ovarian cancer via interferon-gamma, or significantly reduced cytotoxic activity and T cell-related gene expression [6]. An animal study reported that reducing interferon-gamma prolonged prognosis by increasing the intra-tumoral infiltration of CD8-positive T cells [32]. Paradoxically, the maintenance of TILs, cytotoxic activity, and platinum sensitivity in the IR subtype is responsible for the prolonged prognosis. In HGSOC, TILs were an independent prognostic indicator in the R0 + optimal and suboptimal surgery groups. These results and previous findings indicate that TILs may be involved in the response to anticancer drug treatment and radiotherapy via cancer immunity in initial and recurrent treatments.

The present study extracts the histological features and prognosis of HGSOC. One limitation is that the pathogenesis of HGSOC was assessed using surgical specimens at the time of initial treatment. Even in cases assessed as having low lymphocytic infiltration, inflammatory and immune responses might have been strongly induced after the administration of anticancer agents. Alternatively, some cases may have a reduced immune response if bone marrow function was impaired due to the side effects of anticancer agents. It is uncertain whether a similar immune response occurs in cases with a pre-cachexia tendency. It is important to understand that immune responses can be both activated or suppressed depending on the influencing factors and therefore may not be the same as the immune response at surgical removal. A second limitation is the limited number of cases. Comparative studies of the lymphocyte types intraepithelial and stromal could not be performed [33,34]. Detailed studies of residual tumor size and location after suboptimal surgery were difficult. Further case accumulation is needed. A third limitation is that almost no cases were treated with molecularly targeted drugs such as PARP inhibitors or bevacizumab, and BRCA status was unknown. Data from 2018 onwards will require more detailed analysis and case accumulation due to the greater prognostic impact of BRCA status and the addition of maintenance therapy [35]. This is both a limitation and an interesting aspect of this study; before the advent of PARP inhibitors or ICI, when chemotherapy and radiotherapy were the predominant treatment modalities, including for other cancers, the focus of myelosuppression was on neutropenia. However, it is now clear that cancer immunocompetence is maintained by the lymphocyte-based immune cycle. Although the efficacy of ICI in the field of ovarian, fallopian tube, and peritoneal cancer is still unclear, the need for immune maintenance therapy to support lymphocyte-based immunity during chemotherapy, PARP inhibitor, molecular-targeted anticancer agents, or radiotherapy will be discussed in the future. In other cancers, the treatment of cachexia with ghrelin-like agonists and the relationship between blood neutrophil to lymphocyte ratio and treatment prognosis have already been investigated [28,36]. In non-small cell lung cancer, ICI is effective even in the TIL-high group with PD-L1 < 1% [27]. In the IR subtype of HGSOC, maintaining cancer immunocompetence may be effective, and for those in the suboptimal surgery group, ICI in combination with first-line treatment may be a treatment option to improve therapeutic efficacy.

## 5. Conclusions

Intra-tumoral lymphocytic infiltration of the IR subtype is an independent and favorable prognostic indicator in HGSOC and is associated with sensitivity to anticancer agents and radiotherapy via cancer immunocompetence.

## Figures and Tables

**Table 1:** Clinical characteristics of 74 HGSOC cases.

Variables	High TILs	Low TILs	*p*-Value
Number of cases	17	57	
Age (years)	63	61	0.121
median (range)	(48–82)	(34–83)	
Retroperitoneal lymph node sampling	14	39	0.634
Platinum-based chemotherapy			0.622
Neoadjuvant	3	10	
Adjuvant	14	47	
CA125 before initial treatment			0.639
median	574	824	
range	26.0–16,466.0	6.0–17,815.0	
2014 FIGO stage			0.406
I and II	4	10	
III and IV	13	47	
Residual disease			0.092
R (0)	3	11	
Optimal (≤1 cm)	4	25	
Suboptimal (>1 cm)	10	21	

**Table 2:** Pathological characteristics of 74 HGSOC cases.

Variables	High TILs	Low TILs	*p*-Value
Number of cases	17	57	
Architectural grade			0.031
1 and 2	7	40	
3	10	17	
Nuclear grade			0.094
1 and 2	3	4	
3	14	53	
Lymphovascular space invasion			0.532
Present	10	35	
Absent	7	22	
Lymph node metastasis			0.044
Present	9	13	
Absent	5	26	
Intra-tumoral budding			0.319
Present	15	45	
Absent	2	12	
p53 expression status			0.621
Overexpression	12	38	
Complete absence	5	16	

**Table 3:** Clinical data on IR subtypes of high-grade serous ovarian carcinoma in the suboptimal surgery group.

No.	Initial Treatment	CT *	Recurrence-1	Recurrence-2	Recurrence-3	Prognosis
1	Ope + TC6 **	CR	DC6 (2y6m)	Doxil etc (4y3m)		AWD (6y7m)
2	Ope + TC6	CR	RT40Gy (ly)	RT50Gy (3y9m	RT 56Gy (4y10m)	NED (6y10m)
3	Ope + TC6	CR	TC4 (2y5m)	TC2 (7y5m)	Olaparib	NED (9y5m)
4	Ope + TC6	CR				NED (11y3m)
5	Ope +TC4 + IDS + TC4	CR	TC2 + RT (2y2m)	RT (9y2m)		NED (12y4m)
6	Ope + CEP5	CR	RT50Gy (2y)			DOA (3y0m)
7	TC2 + IDS+ TC4	CR	CC2 (7m)	Doxil, Gemcita-bine (ly)		DOD (2y6m)
8	CN4 + IDS + CN6	CR	Progression-1 TC6 (1y3m)	Doxil + CBDCA etc.		DOD (4y4m)
9	TC2 + IDS + TC4	CR	TC6 (3y)	CC2 (4y)		DOD (5y4m)
10	Ope + TC6	CR	TC3 (1y1m)	TC6 (2y5m)	Doxil2 (4y2m), Nogitecan25	DOD (7ylm)

* To estimate the initial treatment effect for computed tomography, ** Cycle numbers are marked to the right of the abbreviation. Ope: Operation, IDS: Interval debulking surgery, RT: Radiotherapy, TC: Paclitaxel + Carboplatin, DC: Docetaxel + Carboplatin, CC: CPT-11 + CBDCA, CN: CPT-11 + NDP, AWD: alive with disease, NED: no evidence of disease, DOA: death of another disease or other, DOD: death of the disease.

**Table 4:** Univariate and multivariate survival time analyses for progression-free survival (PFS) and overall survival (OS) of the 74 cases in high-grade serous ovarian carcinoma. HR: hazard ratio, CI: confidence interval.

Variables	Univariate PFS Analysis		Multivariate PFS Analysis	
	HR (95%CI)	*p*-Value	HR (95%CI)	*p*-Value
Age (<60 years vs. ≥60 years)	1.184 (0.703–1.992)	0.526		
2014 FIGO stage (I and II vs. III and IV)	6.563 (2.350–18.329)	<0.001	2.838 (0.674–11.947)	0.155
CA125 level before initial treatment	1.910 (1.123–3.249)	0.017	1.296 (0.607–2.766)	0.503
Architectural grade (1 and 2 vs. 3)	0.678 (0.390–1.177)	0.167		
Nuclear grade (1 and 2 vs. 3)	1.004 (0.429–2.351)	0.993		
Intra-tumoral budding (presence vs. absence)	3.279 (1.401–7.672)	0.006	1.296 (0.262–6.418)	0.751
Tumor-infiltrating lymphocytes (high TILs vs. low TILs)	2.186 (1.101–4.340)	0.025	2.294 (0.955–5.515)	0.063
Lymphovascular space invasion (presence vs. absence)	2.032 (1.160–3.557)	0.013	1.661 (0.597–4.625)	0.331
p53 expression (overexpression vs. complete absence)	0.975 (0.840–1.133)	0.744		
Lymph node metastasis (presence vs. absence)	1.083 (1.016–1.155)	0.015	0.715 (0.343–1.493)	0.372
R0 + optimal vs suboptimal	2.904 (1.683–5.012)	<0.001	3.330 (1.521–7.294)	0.003
**Variables**	**Univariate** **O** **S Analysis**	***p*** **-** **Value**	**Multivariate** **O** **S Analysis**	***p*** **-** **Value**
	**HR (95%CI)**		**HR (95%CI)**	
Age (<60 years vs. ≥60 years)	0.922 (0.491–1.732)	0.801		
2014 FIGO stage (I and II vs. III and IV)	3.660 (1.126–11.898)	0.031	0.413 (0.076–2.260)	0.308
CA125 level before initial treatment	2.143 (1.100–4.174)	0.025	4.047 (1.290–12.693)	0.017
Architectural grade (1 and 2 vs. 3)	0.532 (0.264–1.071)	0.077		
Nuclear grade (1 and 2 vs. 3)	0.883 (0.313–2.490)	0.815		
Intra-tumoral budding (presence vs. absence)	2.790 (0.989–7.867)	0.052	3.908 (0.732–20.873)	0.111
Tumor-infiltrating lymphocytes (high TILs vs. low TILs)	4.414 (1.552–12.553)	0.005	10.107 (2.337–43.711)	0.002
Lymphovascular space invasion (presence vs. absence)	1.667 (0.844–3.292)	0.141		
p53 expression (overexpression vs. complete absence)	1.087 (0.926–1.267)	0.319		
Lymph node metastasis (presence vs. absence)	1.100 (1.018–1.190)	0.016	0.755 (0.300–1.895)	0.549
R0 + optimal vs suboptimal	2.021 (1.077–3.795)	0.029	4.301 (1.380–13.406)	0.012

## Data Availability

The data presented in this study are available on request from the corresponding authors due to ethical reasons.
